# Prevalence and risk factors of monosymptomatic nocturnal enuresis in Turkish children

**DOI:** 10.4103/0970-1591.65387

**Published:** 2010

**Authors:** Seçil Özkan, Elif Durukan, Elvan Iseri, Serhat Gürocak, Işil Maral, M. Ali Bumin

**Affiliations:** Gazi University Medical Faculty, Department of Public Health, Ankara, Turkey; 1 Gazi University Medical Faculty, Department of Child and Adolescent Psychiatry, Ankara, Turkey; 2 Gazi University Medical Faculty, Department of Urology, Ankara, Turkey

**Keywords:** Enuresis, monosymptomatic, nocturnal

## Abstract

**Objectives:**

To determine the prevalence of primary monosymptomatic nocturnal enuresis (PMNE) and assess risk factors that can cause this disease.

**Methods:**

After the determination of 15 primary schools in the provincial center of Ankara, questionnaires were given to 15,150 students to be answered by their parents. Detailed urologic history was obtained and physical examination applied to the students whose parents answered the questionnaire. After excluding children with polysymptomatic NE, 14060 questionnaires of MNE patients were evaluated. Demographic features with social and medical history of students and their parents, general approach of family to the children, school success of the students and general behavioral attitudes, method of toilet training and the presence of nocturnal enuresis were questioned.

**Results:**

MNE was determined in 9.0% (n: 1266) of the students and nocturnal enuresis frequency was higher in boys than girls (*P*<0.05). Univariate analysis revealed gender, method of toilet training, sleep problems, school success, and general approach of the family to children and general behavioral attitudes of the children as significant factors. In logistic regression analysis; age, male gender, toilette training with threatening method, deep sleeper, sleep walking, being introverted and shy, significantly increases the risk of nocturnal enuresis.

**Conclusions:**

The current study suggests that the methods of toilet training are extremely important to prevent bedwetting and behavioral disorders due to enuresis. Parents should be well-informed about the appropriate toilet training method.

## INTRODUCTION

Enuresis is defined in the DSM-IV as the repeated voiding of urine into bed or clothes at least twice a week for at least three consecutive months in a child who is at least 5 years of age. Nocturnal enuresis (NE) refers to voiding during sleep; diurnal enuresis defines wetting while awake.[1] NE is one of the most common developmental problems among the children.[2] According to the DSM-IV-TR (Diagnostic and Statistical Manual of Mental Disorders-4), secondary reasons such as medicine use (e.g. diuretics), diabetes mellitus, spina bifida and epilepsy must be excluded.[3]

In recent terminology, NE is categorized into two different groups. The first, monosymptomatic NE (MNE), is bedwetting occurring without any day-time incontinence or urological symptoms.[4,5] It might be an explanation of a normal void occurring at an inappropriate and socially unacceptable time and place.[5] In contrast, bedwetting associated with day-time indicators of bladder dysfunction, such as urgency or toileting frequency, is considered polysymptomatic or non-monosymptomatic NE.[6–8] However, the characteristics of different forms of NE have not yet been clarified.

Nocturnal enuresis is an important developmental problem for school age children and it can cause emotional and social problems for the child as well as family.[9] Chronic anxiety, impaired self-esteem, and delayed developmental steps such as attending camps or sleeping at a friend’s house may occur as secondary problems. Frequently, the psychological and developmental damage may actually be more significant and devastating to the child than the symptom of enuresis itself.[10] With this regard, nocturnal enuresis is evaluated as an important public health problem.

The reported prevalence of enuresis at different ages varies considerably because of inconsistencies in the definition of enuresis, differences in the method of data collection, and characteristics of the population sampled. The ratio of the incidence of enuresis in boys vs. girls is 2:1; enuresis is more common at all ages in lower socioeconomic groups and in institutionalized children. The prevalence of nocturnal enuresis among the children older than five years of age was reported as 10-30 % in literature.[9,11] This frequency varies between 9.5 and 13.7 % according to assorted local studies in Turkey.[12]

The aim of this study is to determine the prevalence of MNE among children attending primary schools and investigate the factors affecting monosymptomatic enuresis such as general approach of family to the children, school success of the students and general behavioral attitudes, method of toilet training in the Provincial Center of Ankara.

## MATERIALS AND METHODS

The study was performed in 15 primary schools in the provincial center of Ankara between January 22 and April 15 2003. Ankara, the capital of Turkey, has a population of 3,231,782. There are 884 primary schools in Ankara, with 630,000 students. The sample size was calculated as 13532 students with a population size of 630000, 10% expected frequency and 95% Confidence Interval (Epi Info 6.0). The primary school clusters to provide this sample size were chosen weighting with regard to provincial districts of Ankara. First, three (Çankaya, Yenimahalle and Keçiören) of the eight provincial centers of Ankara were selected by cluster sampling method and weighted by number of students in these provincial centers. Six primary schools from Çankaya, four from Yenimahalle and five from Keçiören were selected by cluster sampling; the questionnaires were given to 15,150 students who attend kindergartens and classes 1 to 5 of these schools to be answered by their parents.

The specific questionnaire was arranged by searching epidemiologic studies about enuresis nocturna from Turkey and other countries.[13,14] It has 45 selected questions in the local language. The questions can be sorted into six categories includes descriptive questions related to child and parents, questions about the general approach of family to the child, the school success of the child, the general behavioral attributes, NE and parasomnia in child. The DSM-IV criteria are used to define children with NE. To differentiate the monosymptomatic NE from polysymptomatic NE, the symptoms which reflect bladder dysfunction such as urgency, frequency, bowel problems were assessed with the questionnaire. Children with polysymptomatic NE were excluded from the study.

The χ^2^ analysis was used in the evaluation of the relation between the presence of nocturnal enuresis and age, sex, school success, the parents’ behavior to the child, the followed method for toilet training, and the behavioral attributes of the child. A logistic regression model was developed to analyze the factors influencing the presence of nocturnal enuresis, the factors found related with nocturnal enuresis in the univariate analysis were put in the model and backward stepwise modeling was used. *P* value under 0.05 was considered “statistically significant”.

## RESULTS

A total of 15100 questionnaires were sent, 14103 (93.1%) were returned. Of the 14,103 questionnaires, 79.3% were answered by the mother, 17.7% by the father and 3% by any other carer of the child at home. MNE was determined in 9.0% of the children (n=1266) where as polisymptomatic NE was found in 0.3% (n=43). After exclusion of children with polysymptomatic NE, 14060 questionnaires of MNE patients were evaluated. Regarding age; 0.9% of children are five to six years old, 18.9% - seven years and 19.6% - eight years. The percentages of 9, 10 and 11 year-old children were 20.6, 19.8 and 20.2% respectively. Seven thousand and sixty (50.2%) children were males and 7000 (49.8%) were female (*P*>0.05).

The univariate analysis of the factors associated with the presence of MNE is shown in Table 1. When the presence of MNE was evaluated according to sex, nocturnal enuresis frequency was found higher in boys than girls (*P*< 0.05). No statistically significant difference was found between right and left hand dominance regarding with nocturnal enuresis (*P*>0.05). Monosymptomatic NE was found 2.24 times more in children toiled trained with threat method and 1.68 times more than the children with award method (95%, CI=1.78-2.81, and 1.27-2.22 respectively).

**Table 1:** Univariate analysis of the factors related with the presence of nocturnal enuresis

	Nocturnal enuresis (%)					
	Present	Absent	χ^2^	*P*	OR	95%CI
Sex						
Female (n=7000)	7,7	92.3			1	–
Male (n=7060)	10,2	89.8	χ2 =27.769	*P*<0.05	1.02	1.01-1.03
Age						
5-6 (n=195)	10,3	89,7			1	–
7(n=2350)	16,1	83.9			1.63	0.83-3.27
8(n=2561)	12.6	87.4			1.27	0.65-2.53
9 (n=2830)	10,6	89.4			1.11	0.57-2.21
10 (n=2792)	7.0	93.0			0.65	0.33-1.30
11 (n=2760)	5.6	94.4			0.59	0.30-1.18
>11 (n=592)	10.5	89.5	χ^2^_*in trend*_ = 169.65	*P*<0.05	0.46	0.23-0.95
Method used for the toilet training						
Awarding (n=8911)	8.0	92.0			1	–
Punishment (n=516)	12.8	87.2			1.68	1.27-2.22
Threatening (n=636)	16.4	83.6			2.24	1.78-2.81
Other (n=3262)	9.8	90.2	χ^2^ =63.201	*P*<0.05	1.24	1.08-1.43
Sleep problem						
Difficulty with falling asleep						
Absent (n=11534)	9.1	90,9			1	–
Present (n=1440)	8.5	91.5	χ^2^ =0.577	*P*>0.05	0.927	0.763-1.127
Sleep talking						
Absent (n=11220)	8.6	91.4			1	–
Present (n=2840)	10.6	89.4	χ^2^ =11.488	*P*<0.05	1.260	1.098-1.446
Sleep bruxism						
Absent (n=11840)	8.7	91.3			1	–
Present (n=2220)	10.6	89.4	χ^2^ =8.715	*P*<0.05	1.236	1.064-1.436
Sleep walking						
Present (n=308)	14.5	85.5			1	
Absent (n=13752)	8.9	91.1	χ^2^=11.742	*P*<0.05	1.724	1.250-2.379
Deep sleeping						
Absent (n=10463)	7.9	92.1			1	–
Present (n=3597)	12.0	88.0	χ^2^ =55.373	*P*<0.05	14.370	12.372-16.690
Waking up in the night						
Absent (n=4903)	12.6	87.4			1	–
Present (n=9154)	7.0	93.0	χ^2^ =120.653	*P*<0.05	1.576	1.391-1.785
School success						
Very successful (n=2430)	7,2	92,8			1	–
Successful (n=7410)	7,9	92.1			0.68	0.40-1.18
Intermediate (n=3355)	11,6	88.4			0.43	0.27-0.69
Low (n=372)	17,2	82,8			0.28	0.18-0.45
Very low (n=116)	23,3	76,7	χ^2^_*in trend*_= 86.602	*P*<0.05	0.26	0.16-0.42
Parents’ behavior towards the child						
Normal (n=644)	8.2	91.8			1	–
Protective (n=8010)	8.7	91.3			1.06	0.79-1.44
Oppressive (n=670)	14.5	85.5			1.89	1.31-2.73
Setting free (n=3874)	8.7	91.3			1.06	0.78-1.46
Indifferent (n=103)	19.4	80,6	χ^2^ = 39.721	*P*<0.05	2.69	1.47-4,88
Children’s general behavioral attributes						
Extroverted (n=2001)	7.0	93.0			1	–
Introverted, touchy (n=1882)	12.1	87.9			1.81	1.44-2.27
Sensitive, can get hurt easily (n=5512)	7.4	92,6			1.05	0.86-1.29
Shy (n=2251)	9.9	90.1			1.45	1.16-1.82
Brawly (n=842)	14.5	85.5			2.24	1.71-2.91
Boorish and cruel (n=163)	13.5	86.5	χ^2^ = 85.946	*P*<0.05	2.06	1.23-3.40

Monosymptomatic NE is more common in children who faced problems with falling asleep, sleep talking, sleep bruxism, sleep walking and deep sleeping as compared to those without these (*P*< 0.05). 41.9 % (n=531) of the children with nocturnal enuresis had experienced a frightening event and 44.7% (n=566) of them had emotional stress (sudden death, birth of new sibling, separation etc.) before the beginning of bedwetting.

Monosymptomatic NE was determined in 33.6 % of the children whose mothers had nocturnal enuresis in their childhood and in 29.3% of the children whose fathers had. The percentage ratios of presence of nocturnal enuresis in the children who have sisters, brothers, relatives from the mother and relatives from the father with nocturnal enuresis in childhood were 20.9%, 25.7%, 25.3% and 25,4% respectively.

The presence of MNE adversely affects success at school in these children (*P*< 0.05). MNE was found more in children whose families have an “oppressing” and “indifferent” behavior towards their children than children whose families have a “normal” behavior according to their self-definitions (OR=1.89; 95% CI=1.31-2.73 and OR=2.69; 95% CI=1.47-4.88 respectively). Furthermore, when the general behavioral attributes of children were analyzed, the children defined as “introvert, touchy, shy, brawly, boorish and cruel” by their families had, respectively, 1.81, 1.45, 2.24 and 2.06 times more MNE than the children defined “extrovert” by their parents [Table 1].

The logistic regression analysis of the factors related with nocturnal enuresis is shown in Table 2. Age, male sex, toilet training with threatening method, sleep talking, sleep walking, deep sleeping, being introverted, touchy, shy or brawly are increasing the risk of having nocturnal enuresis (*P* < 0.05). MNE risk is lower for those with intermediate school success and those who are not successful (*P* < 0.05) (OR=0.615 and 0.422 respectively).

**Table 2:** Logistic regression analysis of the factors related with the presence of nocturnal enuresis

	β	*P*	OR	95%CI
Age	-0.300	<0.05	0.740	0.704-0.777
Sex				
Female			1	—
Male	0.237	<0.05	1.268	1.095-1.468
Toilet training method				
Award			1	—
Punishment	0.321	>0.05	1.378	0.988-1.923
Threat	0.632	<0.05	1.882	1.436-2.467
Other	0.160	>0.05	0.173	0.987-1.395
Sleep talking				
No			1	—
Yes	0.210	<0.05	1.234	1.037-1.468
Sleep walking				
No			1	—
Yes	0.534	<0.05	1.798	1.468-1.982
Deep sleeping				
No			1
Yes	0.707	<0.05	2.029	1.737-2.370
School success				
Successful			1	—
Intermediate	-0.486	<0.05	0.615	0.448-0.844
Not successful	-0.862	<0.05	0.422	0.310-0.574
Children’s general behavioral attributes				
Extroverted			1	—
Introverted, touchy	0.474	<0.05	1.607	1.225-2.109
Sensitive, can get hurt easily	0.141	>0.05	1.152	0.908-1.462
Shy	0.317	<0.05	1.372	1.050-1.794
Brawly	0.673	<0.05	1.960	1.433-2.679
Boorish and cruel	0.356	>0.05	1.427	0.783-2.601

## DISCUSSION

Nocturnal enuresis that has a view of an iceberg is a public health problem that may cause emotional and social problems for both the child and family. Epidemiological studies suggest that the prevalence of NE reduces with age and appears to be independent of culture.[15–19] Yeung *et al*. report that NE is found in 16.1% of five-year-olds, reducing to 10.1% at seven years and 2.2% at 19 years, when the definition of NE was accepted according to DSM-IV.[19] In addition, the prevalence of NE was reported as 9.8-13.7% in other studies performed in Turkey.[9,12,20,21] In our study, the prevalence of MNE was 10.3% in five to six-year-old children and increased to 16.1% in seven-year-old children. The differences might occur due to the definition of NE and evaluating the MNE patients in our study. Nevertheless, MNE prevalence reduced gradually with age, after seven, as the aforementioned study says.

The characteristics of MNE and polysymptomatic NE have not been clarified yet. The importance of appropriate assessment and subsequent treatment of both forms of NE have been described by various authors.[4,22–24] Although indicatives of bladder over activity are frequent day-time voiding, urgency, small urine volumes, variability in the size of the wet patch and low functional bladder capacity,[4,25] some studies suggest that urgency and frequency are the most important symptoms to assess bladder dysfunction.[24,26] In our study, 43 (0.3%) of the children had polysymptomatic NE and those children were assured to admit to the hospital for further evaluation.

When the sex differences were analyzed, nocturnal enuresis was found more in boys than girls, and this is a concordant result with the literature.[3,9,27,28] Although it was reported in various studies that nocturnal enuresis was more frequent in children who were fed on breast milk than the ones did not,[9,29] no statistically significant difference was found between these two groups in our study. Similarly, again being concordant with the literature, no significant relation was determined with left or right hand use dominance.[9] According to a study conducted in Italy, the prevalence of left-handedness was higher in the children with nocturnal enuresis than the control group.[30] Regarding the level of income of family, the less the level of income, the higher frequency of nocturnal enuresis was found. According to a study by Öge *et al*, a statistically significant relation was found between socioeconomic level and nocturnal enuresis.[27] In addition, Ferrara *et al*. reported that nocturnal enuresis was more in children with low socioeconomic level.[30]

There are various pathophysiologic studies aimed for defining the etiology of nocturnal enuresis. Beforehand; studies related to the physiology of sleep, the neurophysiology and the capacity of the urinary bladder, antidiuretic hormone (ADH), and the sodium and potassium concentrations of the urine were evaluated[31,32] but publications implicating the absolute effect of psychological factors are not so many. In the current study, it is considered that nocturnal enuresis is more common among introvert, touchy, shy, brawly, boorish and cruel children than the extrovert ones. Similarly, in a study performed in Belgium, it was reported that the enuretic children have less self-confidence and are more introvert than children who are not enuretic.[33] However, it is still unclear which one is the result; low school success, shy, touchy, and brawly behavior or nocturnal enuresis. Also, the school success of these children is low.[11,33] We observed that better school success lowers the risk of nocturnal enuresis. This result is different but it is acceptable that the children who have a nocturnal enuresis problem might work hard to be successful in school so as not to be stigmatized and ostracized by parents, friends or teachers.

Moreover, the approach of family to the child is an effective factor. In a study performed in Taiwan, nocturnal enuresis was found 1.74 times more in the children who have families with oppressive and authoritarian attitude, 1.6 times more in the children whose families have protective attitude. On the other hand, while the use of the appropriate toilet training method is extremely important, an inappropriate toilet training method is an important risk factor for development of nocturnal enuresis in the child.[22] In the current study, nocturnal enuresis incidence of children who were trained with threatening method was found 2.24 times more than the children who received the rewarding method.

There are publications reporting an important relation between nocturnal enuresis and family history, and genetics.[34–37] In a study performed in Malaysia, positive family history was determined in 53% of the enuretic children.[37] Another study from Turkey declared that this ratio was 63 and 6% in the enuretic and control group, respectively.[35] In our study, 84.4% of the children with nocturnal enuresis were found to have positive family history and nocturnal enuresis was found more in the ones with positive family history. Although genetic factors have an important role in the etiology of nocturnal enuresis, somatic and psychosocial factors have major modulator influence.

## CONCLUSION

The prevalence of MNE is very high and it may cause many developmental problems related to psychological and social aspects. The threat method used in toilet training is more common in Turkish social life. Our study suggested that the methods of toilet training are extremely important to prevent bedwetting and behavioral disorders due to enuresis. The parents should be well-informed about selecting the appropriate method for toilet training.
